# Nocturnal hypoxemia is related to morning negative affectivity in untreated patients with severe obstructive sleep apnea

**DOI:** 10.1038/s41598-022-25842-7

**Published:** 2022-12-08

**Authors:** Hajime Kumagai, Hiroyuki Sawatari, Yuka Kiyohara, Akiko Kanoh, Kana Asada, Kengo Kawaguchi, Aki Arita, Yoko Murase, Noriyuki Konishi, Tetsuro Hoshino, Mitsuo Hayashi, Toshiaki Shiomi

**Affiliations:** 1 Department of Sleep Medicine, Graduate School of Biomedical and Health Sciences, Hiroshima University, Hiroshima, 7348553 Japan; 2 Sleep Disorders Center, Hiroshima University Hospital, Hiroshima, 7348553 Japan; 3 Hiroshima Minato Clinic, Hiroshima, 7340014 Japan; 4 Department of Perioperative and Critical Care Management, Graduate School of Biomedical and Health Sciences, Hiroshima University, Hiroshima, 7348553 Japan; 5 Division of Clinical Support, Hiroshima University Hospital, Hiroshima, 7348553 Japan; 6 Graduate School of Humanities and Social Sciences, Hiroshima University, Higashi-Hiroshima, 7398521 Japan

**Keywords:** Diseases, Medical research

## Abstract

The relationship between sleep apnea and morning affectivity remains unclear. We aimed to clarify how sleep disturbance in patients with obstructive sleep apnea (OSA) influences their affectivity. The enrolled participants underwent the Positive and Negative Affect Schedule on their beds immediately before and after overnight polysomnography. Thirty patients with OSA were divided into two groups according to the apnea–hypopnea index (AHI): mild to moderate OSA (5 ≤ AHI < 30/h) and severe OSA (AHI ≥ 30/h) groups. Additionally, 11 healthy participants (AHI < 5/h) were included as the control group. No independent association was found between affectivity and OSA severity markers in the whole population; however, the severe OSA group had a significantly higher cumulative percentage of sleep time at saturations < 90% (CT90) and worsened morning negative affectivity. Multiple regression analysis showed that CT90 was an independent factor for increasing negative affectivity in the severe OSA group (*p* = 0.0422). In patients with OSA, the receiver operating characteristic curve analysis showed that the best cutoff value for CT90 for predicting no decrease in negative affectivity after sleep was 1.0% (sensitivity = 0.56, specificity = 0.86); the corresponding area under the curve was 0.71. Worsening of negative affectivity in the morning was influenced by nocturnal hypoxemia in patients with severe OSA.

## Introduction

In healthy sleep, we often experience a good night’s sleep and feel refreshed upon awakening. However, for individuals with a destabilized quantity and/or quality of sleep due to sleep deprivation or sleep disorders, this good night's sleep is lost, and may result in non-restorative sleep and sleep inertia.

Obstructive sleep apnea (OSA) is present in 2–34% of the general adult population and is estimated to be present in approximately 1 billion individuals worldwide^1–6^. In patients with OSA, sleep is fragmented by repeated respiratory events, which result in poor sleep quality. As a result, patients with OSA rarely wake up refreshed and, thus, complain of persistent sleepiness, fatigue, depression, and anxiety^1,3,7–10^. Additionally, a more severe OSA may result in more emotionally negative dreams in patients with untreated OSA^11^. Furthermore, intermittent hypoxemia due to OSA causes ischemia–reperfusion injury, which has different effects on the human body compared to chronic hypoxemia^12^. Its effects depend on the severity of hypoxemia, which not only contributes to atherosclerosis, cardiovascular disease, and cognitive decline^13–16^, but may also cause morphological and functional changes in brain neuroimaging studies^17^. Therefore, daily sleep may be disturbed by sleep fragmentation due to obstructive respiratory events in patients with OSA, which may influence their positive and negative affectivity.

In this study, the Positive and Negative Affect Schedule (PANAS) was used to assess subjective affectivity^18^. This is a self-administered questionnaire developed by Watson et al. to assess positive and negative affectivity. The questionnaire has been translated and validated in various languages, including Japanese, and is the most frequently used scale worldwide^19–23^.

To the best of our knowledge, no studies have examined PANAS on the bed immediately before and after nocturnal polysomnography (PSG) to clarify the changes in morning affectivity. Therefore, this study aimed to determine how sleep disturbance influences morning affectivity in patients with untreated OSA. Moreover, we examined the correlation between subjective changes of affectivity in the PANAS and objective polysomnographic indices immediately before and after overnight PSG.

## Results

### Demographics and questionnaires

The participant demographics and questionnaire results are presented in Table 1. A total of 41 participants were included in the study. Of the included participants, 11 participants were in the control group (seven male [63.6%] and four female [36.4%] individuals). The mean age and body mass index (BMI) in the control group were 25.3 ± 14.6 years and 20.3 ± 4.0 kg/m^2^, respectively. The mild to moderate OSA group consisted of 19 patients (15 male [78.9%] and four female [21.1%] individuals), with a mean age of 48.6 ± 12.8 years. The mean BMI was 23.8 ± 3.9 kg/m^2^. In the severe OSA group, all the 11 patients were male, with a mean age of 45.4 ± 14.1 years and a mean BMI of 28.1 ± 4.9 kg/m^2^. There were no significant differences in sex, smoking habits, and co-morbidities, whereas age and BMI were significantly different among the three groups (*p* = 0.0002 and 0.0005, respectively).

**Table 1:** Demographic characteristics and questionnaires.

	Control	Mild to moderate OSA	Severe OSA	*p *value
Number of patients, N	11	19	11	‒
Age, years	25.3 ± 14.6	48.6 ± 12.8	45.4 ± 14.1	0.0002*
Male, N (%)	7 (63.6)	15 (78.9)	11 (100)	0.0961
BMI (kg/m^2^)	20.3 ± 4.0	23.8 ± 3.9	28.1 ± 4.9	0.0005*
Smoking habit, N (%)	1 (9.1)	3 (15.8)	3 (27.3)	0.5155
**Co-morbidity (including duplicates)**				
Hypertension	0 (0)	1 (5.3)	3 (27.3)	0.0653
Diabetes mellitus	0 (0)	0 (0)	2 (18.2)	0.0569
Hyperlipidemia	0 (0)	3 (15.8)	1 (9.1)	0.3715
Others	3 (27.3)	4 (21.1)	3 (27.3)	0.8986
**Questionnaires**				
ESS, points	7.6 ± 6.0	12.6 ± 6.4	10.1 ± 6.0	0.1109
PSQI, points	7.4 ± 4.2	6.7 ± 3.0	6.6 ± 3.7	0.8653
SDS, points	44.0 ± 11.9	41.6 ± 7.4	40.8 ± 9.1	0.6982

AHI, apnea-hypopnea index; BMI, body mass index; ESS, Epworth Sleepiness Scale; OSA, obstructive sleep apnea; PSQI, Pittsburgh Sleep Quality Index; SDS, Self-rating Depression Scale; *, *p* < 0.05.

Regarding the questionnaires, no significant differences were observed in the Epworth Sleepiness Scale (ESS)^24^, Pittsburgh Sleep Quality Index (PSQI)^25^, or Self-rating Depression Scale (SDS)^26^ scores among the three groups (Table 1).

### Polysomnographic data

The mean ± standard deviation (SD) of apnea–hypopnea index (AHI) for the three groups were 1.9 ± 1.3 /h for the control group, 15.9 ± 7.6 /h for the mild to moderate OSA group, and 56.0 ± 23.2 /h for the severe OSA group, showing significant differences (*p* < 0.0001). There were no significant differences in total sleep time (TST) and sleep efficiency among the three groups. Among these groups, there were significant differences in the %stage N1 and N3 (*p* < 0.0001 and *p* = 0.0056, respectively), but not in the %stage N2 and rapid eye movement (REM). Additionally, the arousal index, minimum saturation of percutaneous oxygen, and cumulative percentage of time at saturations < 90% during sleep (CT90) were significantly different among the three groups (*p* = 0.0097, < 0.0001, and < 0.0001, respectively), but not the periodic limb movement (PLM) index (Table 2).

**Table 2:** Polysomnographic data.

	Control	Mild to moderate OSA	Severe OSA	*p* value
TST (min)	492.7 ± 113.1	465.5 ± 139.9	493.5 ± 87.6	0.7684
Sleep efficiency (%)	82.5 ± 17.8	83.4 ± 11.4	80.3 ± 11.4	0.8293
Stage N1 (%)	10.9 ± 4.4	17.8 ± 9.3	34.3 ± 15.9	< 0.0001*
Stage N2 (%)	46.0 ± 11.8	51.1 ± 12.9	45.0 ± 13.8	0.3856
Stage N3 (%)	13.6 ± 8.7	6.8 ± 6.7	4.1 ± 4.2	0.0056*
Stage REM (%)	14.4 ± 7.4	17.9 ± 6.8	15.2 ± 4.7	0.3054
AHI (/h)	1.9 ± 1.3	15.9 ± 7.6	56.0 ± 23.2	< 0.0001*
Arousal index (/h)	15.9 ± 7.4	17.7 ± 9.0	37.1 ± 19.2	0.0097*
Min SpO_2_ (%)	92.2 ± 1.7	87.4 ± 4.7	73.1 ± 11.0	< 0.0001*
CT90 (%)	0.0 ± 0.0	0.1 ± 0.3	11.7 ± 12.6	< 0.0001*
PLM index (/h)	3.3 ± 4.5	1.4 ± 2.3	2.3 ± 3.4	0.3475

AHI, apnea-hypopnea index; CT90, percentage of cumulative time of blood oxygen saturation below 90% during sleep; Min SpO_2_, minimum saturation of percutaneous oxygen; OSA, obstructive sleep apnea; PSG, polysomnography; PLM, periodic limb movement; TST, total sleep time; *, *p* < 0.05.

### The PANAS data

**Figure 1:**
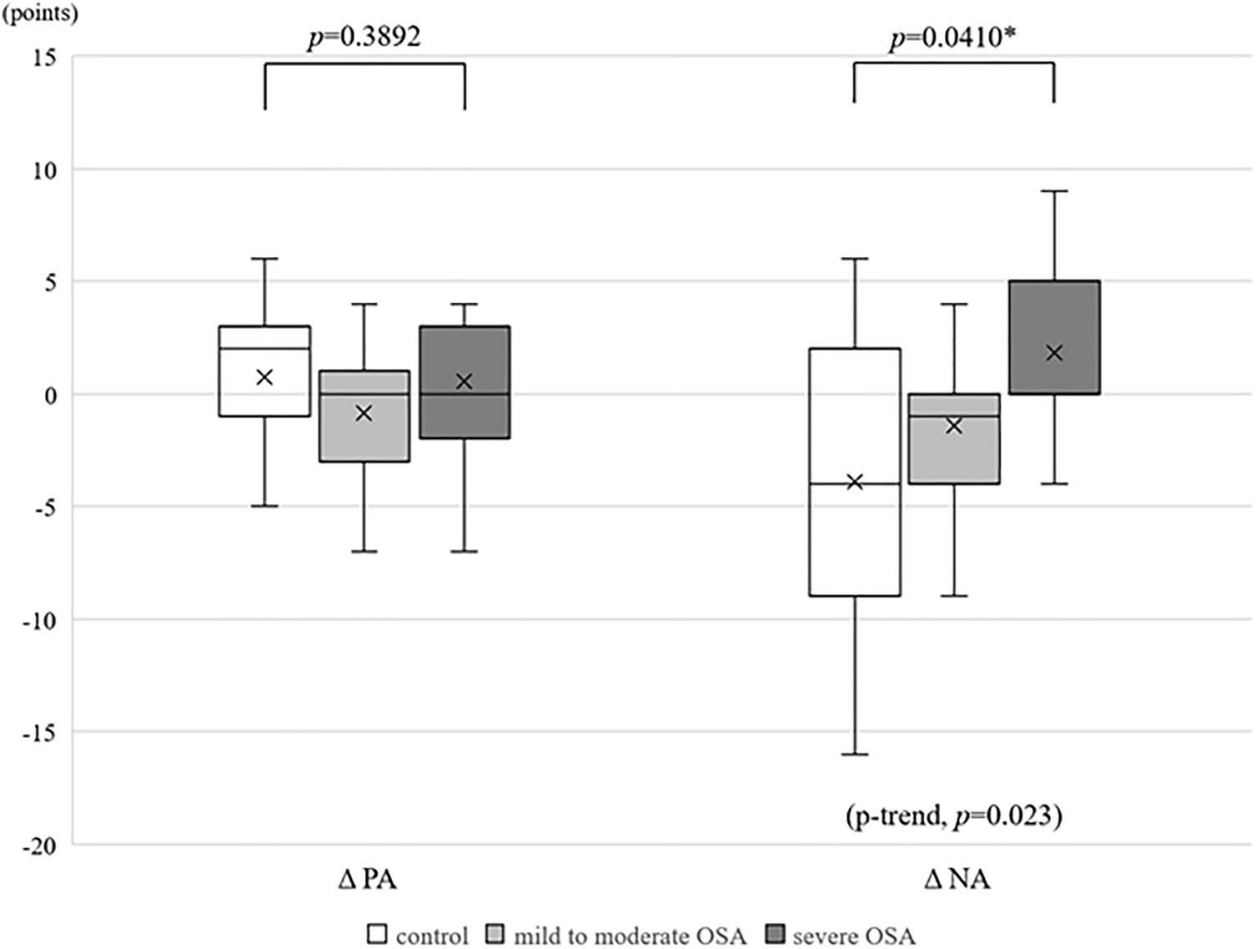
Changes in positive and negative affectivity in the PANAS. A significant difference was observed between the three groups only in Δ NA (*p* = 0.0410). There was a significant trend for Δ NA to increase as OSA became more severe (p-trend, *p* = 0.023). OSA, obstructive sleep apnea; PA, positive affectivity; PANAS, positive and negative affect schedule; NA, negative affectivity; Δ PA, positive affect score on awakening minus positive affectivity score before sleep; Δ NA, negative affect score on awakening minus negative affectivity score before sleep, *: *p* < 0.05.

In each of the three groups, a comparison of the PANAS scores immediately before and after PSG showed no significant differences in either positive affectivity (PA) or negative affectivity (NA) (Table 3A). For positive affect score on awakening minus positive affect before sleep (Δ PA), there was no significant difference among the three groups, whereas a significant difference was observed in the negative affect score on awakening minus negative affect score before sleep (Δ NA) (*p* = 0.0410) (Table 3B). For Δ NA, a trend of increasing Δ NA with increasing AHI was observed (p‒trend, *p* = 0.023) (Fig. 1).

**Table 3:** PANAS scores immediately before and after overnight PSG.

	Control			Mild to moderate OSA			Severe OSA		
	Before PSG	After PSG	*p* value	Before PSG	After PSG	*p* value	Before PSG	After PSG	*p* value
**(A)**									
PA, points	23.1 ± 10.3	23.8 ± 10.1	0.7009	25.3 ± 9.1	24.5 ± 8.6	0.2146	20.6 ± 9.9	21.2 ± 10.1	0.7031
NA, points	22.6 ± 10.0	18.7 ± 7.3	0.0951	22.4 ± 9.7	20.9 ± 10.3	0.1557	16.3 ± 6.3	18.1 ± 8.7	0.1377

	Control	Mild to Moderate OSA	Severe OSA	*p *value					
**(B)**									
ΔPA, points	0.7 ± 6.1	− 0.8 ± 2.9	0.5 ± 4.6	0.3892					
ΔNA, points	− 3.9 ± 7.0	− 1.4 ± 4.2	1.8 ± 3.7	0.0410*					

AHI, apnea–hypopnea index; NA, negative affect in the PANAS; OSA, obstructive sleep apnea; PA, positive affect in the PANAS; PANAS, positive and negative affect schedule; PSG, polysomnography.

### Associated factors for the positive and negative affectivity in the PANAS

**Figure 2:**
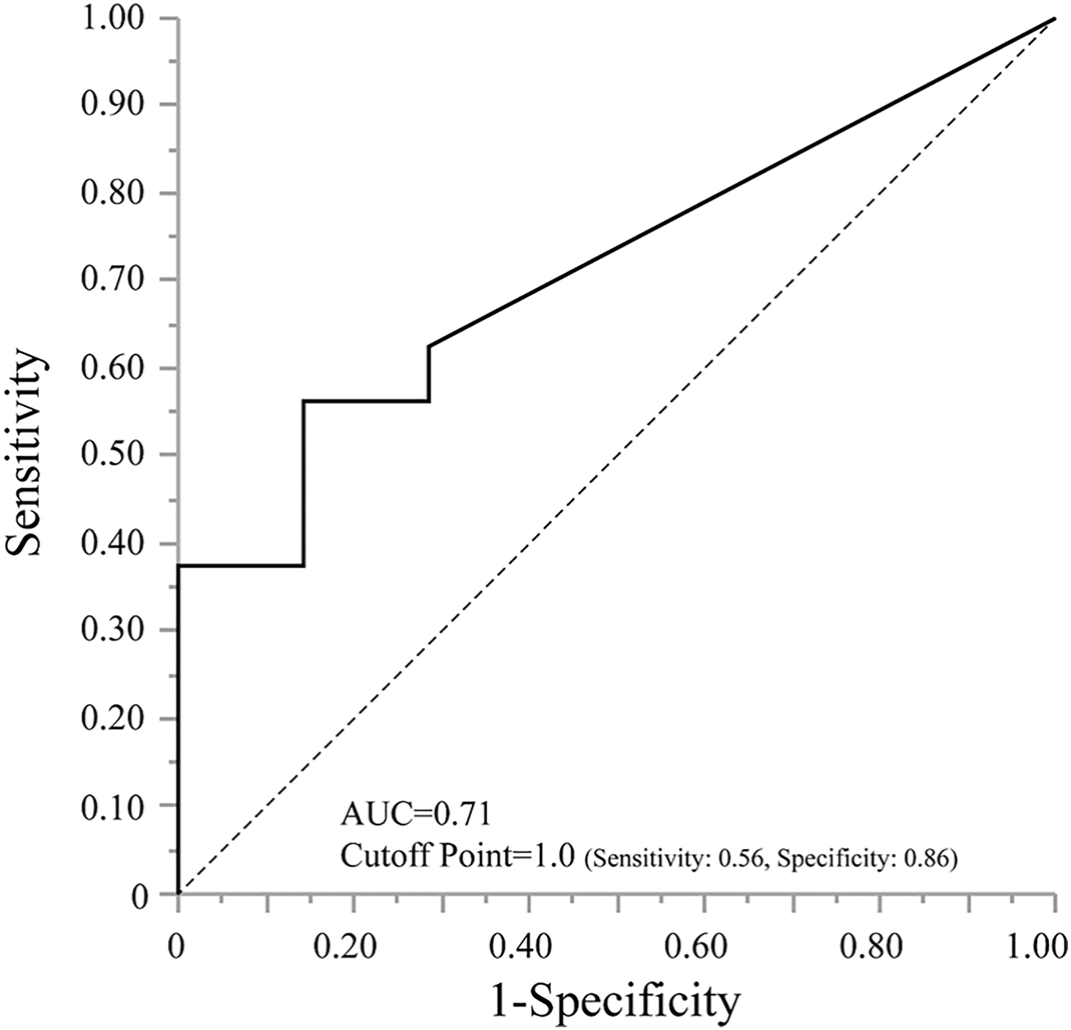
The ROC curve for CT90. The ROC curve analysis showed that the best cutoff value for CT90 for predicting increase or unchanged in negative affectivity after sleep was 1.0% (sensitivity, 56.3%; specificity, 85.7%), the corresponding AUC was 0.714. AUC, corresponding an area under the curve; CT90, percentage of cumulative time of blood oxygen saturation below 90% during sleep; ROC, receiver operating characteristic.

Univariate analysis was performed for PSG-related factors against Δ PA and Δ NA. For Δ PA, no significant PSG-related factors were found in any of the groups. In contrast, for Δ NA, SDS in the control group (r = − 0.7868, *p* = 0.0041), TST in the mild to moderate OSA group (r = − 0.5193, *p* = 0.0227), and AHI and TST in the severe OSA group (r = 0.6109, *p* = 0.0458, r = − 0.7399, *p* = 0.0092, respectively) were significantly correlated with Δ NA. In multivariate analysis for Δ NA in all groups, no significant factors were observed both before and after adjustment for age and BMI (Table 4A). However, when subsequently analyzed in each group, adjusted for age and BMI, although multivariate analysis for Δ NA showed that there were no significant associated factors in the two groups other than the severe OSA group, CT90 was an independent and significant associated factor for Δ NA in the severe OSA group (standardized β = 1.4504, *p* = 0.0422) (Table 4B). Regarding the receiver operating characterisric (ROC) curve analysis, the best cutoff value of CT90 for predicting no decrease in NA after sleep in patients with OSA was 1.0%, with sensitivity 0.56 and specificity 0.86. The corresponding area under the curve (AUC) was 0.71 (Fig. 2).

**Table 4:** Multivariate analysis in associated factors for Δ Negative Affect in the PANAS.

	All (unadjusted)		All (adjusted)			
	standardized β	*p *value	standardized β	*p *value		
**(A)**						
Age	‒	‒	0.0156	0.9358		
BMI	‒	‒	− 0.1301	0.5487		
TST	− 0.2118	0.1838	− 0.1867	0.3261		
AHI	0.5718	0.0858	0.6515	0.0904		
ArI	− 0.1274	0.5625	− 0.1459	0.5241		
Min SpO_2_	0.2392	0.3148	0.2583	0.3179		
CT90	0.1338	0.5803	0.1784	0.5292		
PLMI	− 0.1196	0.437	− 0.114	0.4713		

	Control		Mild to Moderate OSA		Severe OSA	
	standardized β	*p* value	standardized β	*p* value	standardized β	*p* value
**(B)**						
Age	− 0.3329	0.706	− 0.0236	0.948	− 0.1460	0.5737
BMI	− 0.2995	0.7711	0.0093	0.9839	− 1.3870	0.1280
TST	− 0.7951	0.4878	− 0.7368	0.9839	− 0.6648	0.1326
AHI	0.3114	0.7719	0.0933	0.7381	0.5456	0.4562
Arousal index	− 0.5488	0.5338	0.1222	0.6848	− 0.2102	0.4825
Min SpO_2_	0.3316	0.7162	0.0005	0.9988	0.6358	0.0961
CT90	0	‒	− 0.5191	0.3316	1.4504	0.0422*
PLMI	− 0.2449	0.8126	0.2603	0.6388	0.3052	0.1389

AHI, apnea–hypopnea index; BMI, body mass index; CT90, percentage of cumulative time of blood oxygen saturation below 90% during sleep; Min SpO_2_, minimum saturation of percutaneous oxygen; OSA, obstructive sleep apnea; PANAS, positive and negative affect schedule; PLM, periodic limb movement; TST, total sleep time; *, *p* < 0.05.

## Discussion

We assessed the changes in positive and negative affectivity using the PANAS immediately before and after overnight PSG and found that NA increased after nocturnal sleep in patients with severe OSA. Sleep disturbance in the severe OSA group had a stronger impact on NA rather than on PA. Furthermore, there was a significant independent impact of CT90 on the increase of NA after sleep, suggesting that sleep may intensify NA in untreated patients with severe OSA who are exposed to hypoxemia every night. The results also showed that NA on awakening was unlikely to be less than NA before sleep when CT90 was > 1% of TST, suggesting that this may influence morning affectivity and sleep inertia in patients with OSA.

To date, most studies examining associations between affectivity and disease have been conducted using either subjective assessments with questionnaires or objective assessments, such as functional magnetic resonance imaging (fMRI)^27–31^. In contrast, to the best of our knowledge, no studies have simultaneously assessed the association between changes in PA and NA immediately before and after overnight PSG and PSG indices, as we have made in the present study. This method was considered useful for assessing the relationship between sleep quality and changes in PA and NA.

The PANAS is a method for assessing positive and negative affectivity and is structured from the standpoint that the two factors, positive and negative affectivity, are independent of each other. Each of the 10 terms selected for each factor was devised to have only one meaning; thus, positive and negative affectivity can be assessed efficiently with high reliability. Additionally, PANAS is sensitive to fluctuations in positive and negative affectivity when assessed using short-term instructions, such as right now or today^18^. In this assessment method, PA of the PANAS is related to activity, attention, and pleasure, while NA is related to stress, anxiety, and discomfort, among others.

In the present study, patients filled out the PANAS on the bed immediately before and immediately after PSG, which may have allowed us to assess sleep-induced changes in affectivity as quickly as possible, without retrospective bias. The results showed that the NA on awakening significantly increased in the severe OSA group compared to the NA before sleep, with significant differences among the three groups. Conversely, PA did not change significantly among the three groups before and after PSG. This suggests that nocturnal sleep disturbance in the severe OSA group was more likely to influence NA rather than PA.

Sleep inertia refers to the prolonged sleepiness, lack of refreshment, and temporary cognitive disturbance that occur during the transition from sleep to wakefulness^32–34^. Sleep inertia is a characteristic of sleep deprivation and central hypersomnia, especially idiopathic hypersomnia. Sleep inertia may also occur in patients with severe OSA. Although the mechanism of sleep inertia has not yet been fully elucidated^32^, there are several hypotheses, including the hypothesis that sleep inertia is caused by awakening before adenosine is completely eliminated or by delayed reactivation of brain regions^33,34^. Based on these hypotheses, sleep may be fragmented by frequent obstructive respiratory events owing to severe OSA, and the quality and quantity of sleep may be reduced. This can result in arousal before adenosine is completely eliminated, thus, delaying brain reactivation and creating sleep inertia. In the present study, the severe OSA group had increased NA after sleep, suggesting that increasing NA may have influenced sleep inertia. As the relationship between affectivity and sleep inertia is still largely unexplored, further research is required.

The following are the possible reasons for the increase of NA. Sleep is fragmented due to frequent obstructive respiratory events caused by severe OSA; thus, affectivity is not reset during sleep when it should be^35^. In the severe OSA group, prolonged exposure to hypoxemia during sleep may lead to inadequate rest in the amygdala during sleep, which is involved in affectivity control, thereby leading to inadequate affectivity control and recovery and increased NA. Furthermore, depression and anxiety in patients with OSA are associated with damage to the brain regions involved in affectivity, such as the amygdala and hippocampus^36,37^. Selective damage to the amygdala and morphological changes in the hippocampus and gray matter have been noted, especially in patients with severe OSA^17,38^. In addition, unlike the oxygen desaturation index (ODI) and hypoxic burden^39^, CT90 is a marker that reflects sustained hypoxemia, which includes OSA, obesity, and chronic obstructive pulmonary disease. These would support the findings that CT90 was a significant factor in the increase of NA, NA was unlikely to decrease after sleep when CT90 is > 1% of TST in the severe OSA group, and patients with chronically accumulated sleep debt had an increased amygdala response to negative affectivity^30^. These findings suggest that, in the severe OSA group, it is crucial to manage sleep-disordered breathing and improve repeated obstructive respiratory events during sleep to prevent increasing NA after sleep. The cutoff value of 1.0% for CT90 calculated from ROC analysis means that among the 8-h sleep duration per day, the sleep time with blood oxygen saturation < 90% would be approximately ≥ 5 min. Moreover, if hypoxemia lasts for > 5 min, the brain, which is involved in affectivity, may be damaged. This suggests that in patients with severe OSA, managing CT90 to < 1% may be an indicator for managing OSA so that NA is not exacerbated by sleep. Furthermore, the indicator may motivate patients with severe OSA to continue CPAP therapy.

This study had several limitations. As it was a retrospective study of Japanese individuals only, the possibility of selection bias owing to differences in race and customs cannot be ruled out. Additionally, the possibility that there were false declarations of affectivity cannot be ruled out; it was difficult to confirm the truth of the declarations because affectivity is something that only the patients themselves know. However, as the patients filled out the PANAS on the bed just before bedtime and immediately after awakening, we believe that retrospective bias is unlikely to have occurred and that the PANAS was filled out as accurately as possible. Affectivity is complex, with numerous factors involved; thus, the assessment of affectivity is not something that can be done solely with the PANAS. As the sample size in this study was small, we would like to examine this issue with a larger number of patients in the future. In addition, it may be necessary to assess the impact of multiple perspectives using various indices, such as fMRI evaluation. In the severe OSA group, no significant difference was observed in NA immediately before and after PSG. This may be attributed to the small sample size. Moreover, it could be attributed to the fact that some cases of patients with apnea or hypopnea predominance, even those having the same OSA severity, as well as the mixture of these cases, may have affected hypoxemia and, therefore, may have reflected in NA. It is also possible that many patients in the control and mild to moderate OSA groups had a CT90 of 0%, making it difficult to accurately assess the relationship with NA. Therefore, in the future, it may be necessary to evaluate changes in NA using AHI and other indices, such as ODI and hypoxic burden. Future studies are also needed to determine whether the treatment of hypoxemia due to severe OSA improves NA, which is increased by sleep, or inhibits sleep inertia.

In patients with suspected OSA, the PANAS was performed immediately before and after overnight PSG to assess the changes in PA and NA. In patients with severe OSA, the impact on NA was greater than that on PA, and NA increased on awakening than before nocturnal sleep. It was also found that the NA increased according to the time of patient’s exposure to hypoxemia. Our results showed that hypoxemia in severe OSA was significantly associated with morning exacerbation of negative affectivity.

## Methods

### Participants

**Figure 3:**
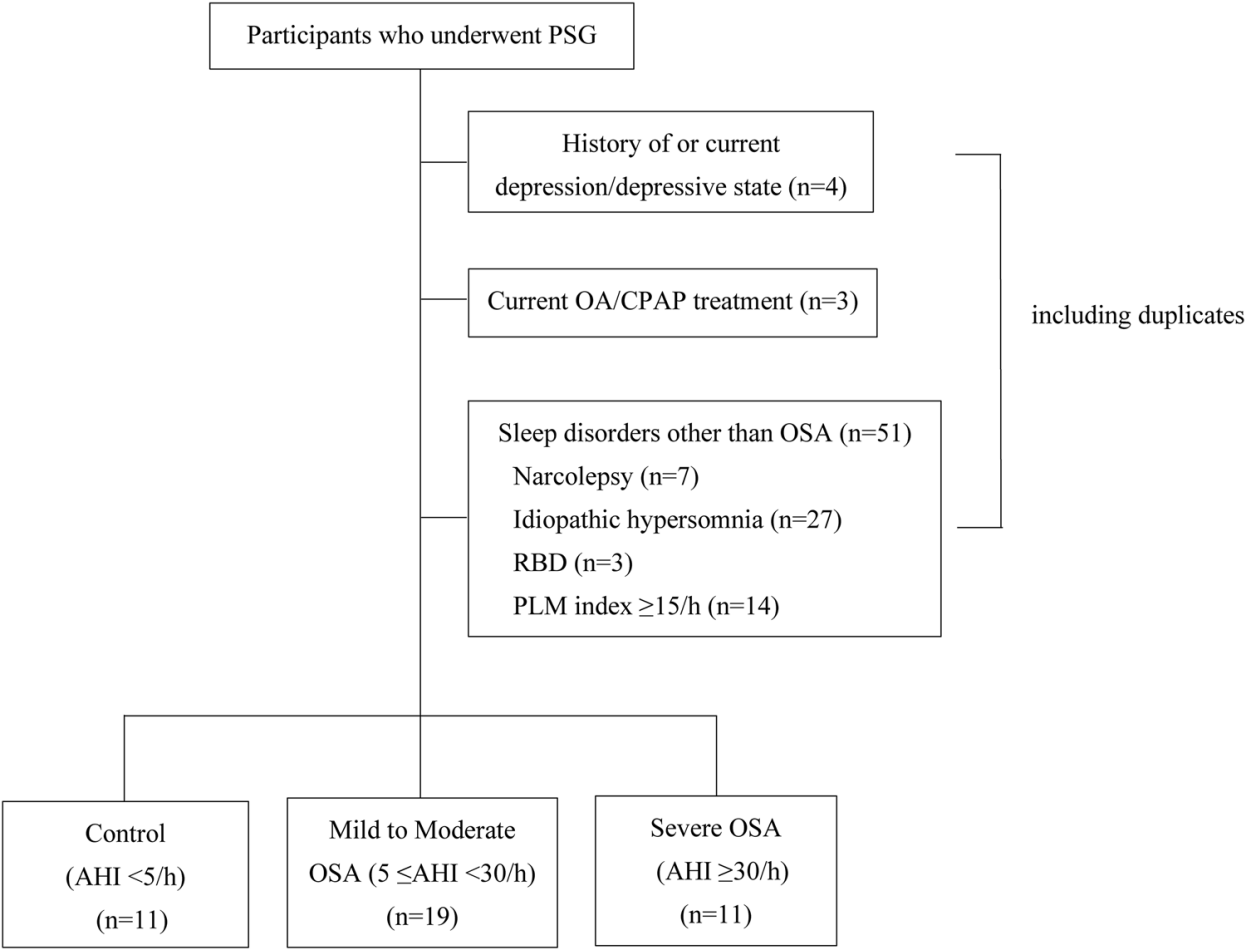
Flow diagram of this study. AHI, apnea-hypopnea index; CPAP, continuous positive airway pressure; OA, oral appliance; OSA, obstructive sleep apnea; PLM, periodic limb movement; PSG, polysomnography; RBD, rapid eye movement sleep behavior disorder.

This study included 89 participants with suspected OSA who visited Hiroshima University Hospital Sleep Medicine Center and Hiroshima Minato Clinic between June 2021 and June 2022 and underwent PSG and self-administered questionnaires. The exclusion criteria were narcolepsy, idiopathic hypersomnia, rapid eye movement sleep behavior disorder, periodic limb movement index ≥ 15/h, previous or current treatment for depression, oral appliances, or continuous positive airway pressure on OSA treatment (including duplication). Participants with OSA were divided into two groups according to the AHI: mild to moderate OSA (5 ≤ AHI < 30) and severe OSA (AHI ≥ 30) groups. In addition, healthy participants with AHI < 5 were included as a control group in this study (Fig. 3). Comparisons were made among the three groups regarding subjective ratings using self-administered questionnaires and PSG indices.

This study was conducted in accordance with the Declaration of Helsinki and approved by the Institutional Review Board of Hiroshima University (approval numbers: E‐2684, November 22, 2021; E2022‐0027, May 24, 2022). This was a retrospective cross-sectional observational study; therefore, an opt-out statement was used for participant consent. Informed consent was obtained from all participants.

### Questionnaires

At the initial visit, the participants were administered the ESS^24^, PSQI^25^, and SDS^26^, which the participants completed on their own in addition to recording their age and sex, and having their BMI measurements taken. Daytime sleepiness was rated on an ESS score of 0–24 points, with higher ESS values indicating greater daytime sleepiness; an ESS score of ≥ 11 points was considered excessive daytime sleepiness. Sleep quality was also assessed using the PSQI; a higher PSQI value indicated poorer sleep quality. Depression was assessed using the SDS, with higher SDS scores indicating a more severe depressive state. Positive and negative affectivity were assessed using the Japanese version of the PANAS^19^, which was self-administered by participants on the bed immediately before and after overnight PSG. Positive and negative affectivity on the PANAS were defined as PA and NA, respectively. The PANAS consists of 10 questions on both PA and NA, which are answered on a 6-point scale, and the total score is used to assess affectivity (Fig. S1 in the supplemental material). Higher total scores for PA or NA indicate stronger affectivity. The Δ PA was defined as the PA score immediately after PSG minus the PA score immediately before PSG, and Δ NA was defined as subtracting a NA score immediately before PSG from a NA score immediately after PSG. The PANAS is commonly used in addition to the medical interview in a variety of conditions and diseases, including cancer, heart disease, and fibromyalgia^27–29^.

### Sleep study

**Figure 4:**
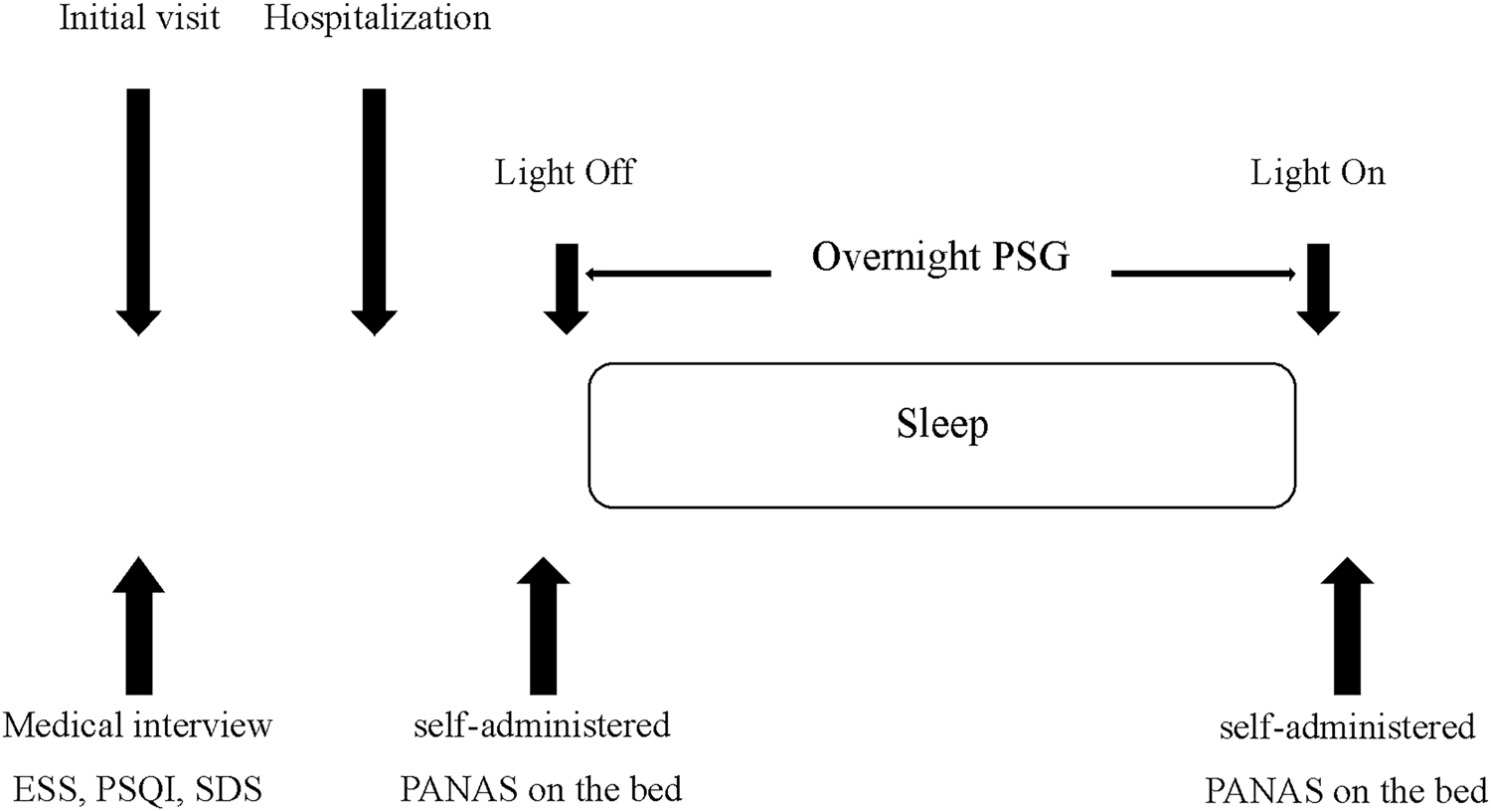
Study protocol. The protocol for this study was as follows: Participants were interviewed at the initial visit and self-administered the ESS, PSQI, and SDS. They also self-administered the PANAS immediately before and after sleep using PSG. ESS, Epworth Sleepiness Scale; PANAS, positive and negative affect schedule; PSG, polysomnography; PSQI, Pittsburgh Sleep Quality Index; SDS, Self-rating Depression Scale.

A PSG was performed using PSG-1100 (NIHON KOHDEN, Tokyo, Japan) and Somnotouch‒RESP (Somnomedics, Randersacker, Germany). Electroencephalogram (EEG), electrooculogram, electromyogram of the submental and anterior tibial muscles, electrocardiogram, nasal flow, chest and abdominal movements with respiratory effort, oxygen saturation, snoring, and body position data were recorded. The PSG data were manually analyzed by certified sleep technologists who were blinded to patient information based on the American Academy of Sleep Medicine scoring criteria (version 2.3 and 2.5). Hypopnea was defined as either arousal or a 30% decrease in airflow for at least 10 s with 3% oxygen saturation. The AHI was calculated as the average number of apnea and hypopnea episodes per hour during sleep. Sleep efficiency was calculated as the ratio of TST on the EEG to time in bed. The %stage N1, N2, N3, and REM indicate the percentage of sleep stages N1, N2, N3, and REM in the TST. The arousal and PLM indices were defined as the number of events per hour. Finally, CT90 was the calculated percentage of the cumulative time of blood oxygen saturation below 90% during TST. The study protocol is shown in Fig. 4.

### Statistical analysis

All statistical analyses were performed using JMP v.16.2.0 (SAS Institute Japan, Tokyo, Japan). Descriptive statistics are expressed as means ± SDs and number of participants (%). To compare the differences among the three groups, Tukey–Kramer’s HSD test and the Wilcoxon/Kruskal–Wallis test were used after testing for normal distribution. Additionally, the p‒trend for Δ NA among the three groups was tested using the Jonckheere–Terpstra test. To compare the differences before and after PSG with respect to the PA and NA scores, paired t-tests were conducted for continuous variables. For univariate regression analysis, Spearman’s rank or Pearson’s correlation coefficients were estimated. Multiple regression analysis was used to estimate factors associated with Δ NA. The ROC curve analysis was used to evaluate the best cutoff value of CT90 to predict no decrease in negative affectivity after sleep in the participants with OSA. We performed a Youden test to determine the best cut-off. All comparisons were two-tailed, with a *p* value of < 0.05 considered statistically significant.

## Supplementary Information

Supplementary Information.

## Data Availability

The datasets used and analysed during the current study available from the corresponding author on reasonable request.
