# Supplementary material for: Nocturnal hypoxemia is related to morning negative affectivity in untreated patients with severe obstructive sleep apnea
Source: Sci Rep. 2022 Dec 8;12:21262. doi: 10.1038/s41598-022-25842-7 (PMC9732184; doi:10.1038/s41598-022-25842-7)
Supplement: Supplementary file 1 — Supplementary Information. [file 41598_2022_25842_MOESM1_ESM.pdf]

Nocturnal hypoxemia is related to morning negative affectivity in untreated patients with severe obstructive sleep apnea

Hajime Kumagai [1,2,3*], Hiroyuki Sawatari [4], Yuka Kiyohara [1,2], Akiko Kanoh [5], Kana Asada [5], Kengo Kawaguchi [1], Aki Arita [1], Yoko Murase [1], Noriyuki Konishi [1], Tetsuro Hoshino [1], Mitsuo Hayashi [6], and Toshiaki Shiomi [1,2]

[1] Department of Sleep Medicine, Graduate School of Biomedical and Health Sciences, Hiroshima University, Hiroshima 7348553, Japan

[2] Sleep Disorders Center, Hiroshima University Hospital, Hiroshima 7348553, Japan

[3] Hiroshima Minato Clinic, Hiroshima 7340014, Japan

[4] Department of Perioperative and Critical Care Management, Graduate School of Biomedical and Health Sciences, Hiroshima University, Hiroshima 7348553, Japan

[5] Division of Clinical Support, Hiroshima University Hospital, Hiroshima 7348553, Japan

[6] Graduate School of Integrated Arts and Sciences, Hiroshima University, Higashi-Hiroshima 7398521, Japan

Supplementary Fig. S1 The PANAS questionnaire

This questionnaire consists of several words that describe different feelings and emotions. Please read each item and then circle your appropriate current affect from 1: not at all, 2: very slightly, 3: a little, 4: moderately, 5: quite a bit, 6: extremely.

This consists of 10 items each of positive and negative affect and is evaluated using a 6–point scale. The total score for each affect was used to evaluate the intensity of the affect.

1	Nervous	1	2	3	4	5	6
2	Active	1	2	3	4	5	6
3	Afraid	1	2	3	4	5	6
4	Proud	1	2	3	4	5	6
5	Upset	1	2	3	4	5	6
6	Scared	1	2	3	4	5	6
7	Strong	1	2	3	4	5	6
8	Excited	1	2	3	4	5	6
9	Jittery	1	2	3	4	5	6
10	Determined	1	2	3	4	5	6
11	Distressed	1	2	3	4	5	6
12	Inspired	1	2	3	4	5	6
13	Alert	1	2	3	4	5	6
14	Enthusiastic	1	2	3	4	5	6
15	Ashamed	1	2	3	4	5	6
16	Irritable	1	2	3	4	5	6
17	Interested	1	2	3	4	5	6
18	Guilty	1	2	3	4	5	6
19	Hostile	1	2	3	4	5	6
20	Attentive	1	2	3	4	5	6
